# Predictive Value of the Cally Score in Determining Surgical Strategy for Complicated Left-Sided Colonic Diverticulitis: A Retrospective Cohort Study

**DOI:** 10.3390/medicina61081455

**Published:** 2025-08-13

**Authors:** Feyyaz Gungor, Huseyin Kılavuz, Muhammed Furkan Arslan, Murat Demir, Yusuf Yunus Korkmaz, Ali Bekraki, Idris Kurtulus

**Affiliations:** Department of General Surgery, Basaksehir Cam and Sakura City Hospital, Istanbul 34480, Turkey; drhuseyinkilavuz@gmail.com (H.K.); drmuhammedfurkanarslan@gmail.com (M.F.A.); muratdemir57@gmail.com (M.D.); yusufyunuskorkmaz@gmail.com (Y.Y.K.); abekraki@hotmail.com (A.B.); idriskurtulus@gmail.com (I.K.)

**Keywords:** complicated diverticulitis, Hartmann procedure, primary anastomosis, CALLY index, Prognostic Nutritional Index, HALP score, mGPS, albumin, surgical decision-making

## Abstract

*Background and Objectives*: Complicated left-sided colonic diverticulitis is one of the important causes of hospital admissions and emergency surgery in industrialized societies and requires serious clinical decision-making processes for patient management. This study aims to evaluate the predictive role of albumin-based nutritional indices in deciding on surgical strategy (primary anastomosis vs. Hartmann procedure) in patients treated operatively for complicated left-sided colonic diverticulitis. *Materials and Methods*: This retrospective single-center study included 57 patients who were operatively treated for Hinchey stage III–IV diverticulitis between 2021 and 2024. Patients were divided into two groups according to surgical method: Hartmann procedure (n = 40) and primary anastomosis (n = 17). Prognostic Nutritional Index (PNI), Hemoglobin–Albumin–Lymphocyte–Platelet Score (HALP), CRP–Albumin–Lymphocyte (CALLY) Index, and Modified Glasgow Prognostic Score (mGPS) were evaluated as albumin-based nutritional indices in the preoperative period. Predictive parameters were determined using ROC analysis and multivariate logistic regression. *Results*: Albumin level, PNI, HALP, and CALLY scores were found to be significantly lower in the Hartmann procedure group. Additionally, the proportion of patients with mGPS score 2 was significantly higher in the Hartmann procedure group (57.5% vs. 5.9%; *p* < 0.001). In the ROC analysis, the cut-off value for the CALLY index was determined as 0.45 (AUC: 0.826). In multivariate analysis, albumin < 38.5 g/L (OR: 16.53), CALLY index < 0.45 (OR: 6.40), and systemic inflammatory response syndrome (SIRS) detection (OR: 12.98) were determined as independent factors predicting the Hartmann procedure. *Conclusions*: A low CALLY index was found to be independent predictors for the Hartmann procedure. The CALLY index, which reflects the inflammatory response, immune capacity and nutritional status, can assist surgeons in making objective, and individualized decisions by holistically evaluating the patient’s physiological status. Multicenter prospective studies are required to confirm the clinical validity of the findings.

## 1. Introduction

Colonic diverticular disease is a major cause of hospital admissions and imposes a significant burden on healthcare costs in Western and industrialized societies. Each year in the United States (US), there are more than 200,000 hospitalizations and nearly 300,000 emergency department visits due to diverticular disease, costing more than USD 2 billion in additional annual healthcare expenditures [1,2]. In Western countries, the majority of patients presenting with diverticular disease are diagnosed with left-sided colonic diverticulitis. Symptomatic diverticulitis occurs in approximately 4–5% of individuals with diverticulosis, and cases of complicated diverticulitis account for 10–20% of hospitalizations, representing a significant burden on surgical services [3,4]. Although the management of acute complicated diverticulitis is increasingly conservative, the approach is multifaceted and varies depending on disease severity, patient characteristics, and available hospital resources [5]. It is reported that approximately 15% of patients admitted to the hospital due to colonic diverticulitis require operative treatment [6].

The operative treatment methods for complicated left-sided colonic diverticulitis cover a wide range, from conventional surgery to robotic surgery [7]. Surgical techniques vary depending on the severity of the disease and patient-specific factors and include options such as drainage only, primary anastomosis (with or without a diverting ileostomy), or the Hartmann procedure [8]. Although various surgical techniques have been described, the current literature suggests that the Hartmann procedure is performed in more than 90% of patients treated operatively for complicated left-sided colonic diverticulitis [9,10]. The Hartmann procedure is considered a life-saving technique frequently preferred in patient-specific high-risk clinical situations and surgically challenging cases. However, this approach negatively affects patient quality of life and significantly burdens the healthcare system. Although it provides a technical solution for the surgical team, it creates significant disadvantages both at the individual level and in terms of the healthcare system when evaluated from a patient-centered perspective.

Additionally, the literature reports that a significant proportion of end colostomies—approximately 30%—are not reversed [11]. Therefore, patients should be evaluated carefully before the Hartmann operation, and unnecessary end colostomy should be avoided. In this context, predictive parameters that can be used in the preoperative period during the surgical decision-making process may contribute to surgeons correctly determining the appropriate strategy and providing realistic counseling to patients.

In recent years, the utility of objective biomarkers—especially composite indices that reflect inflammation, immune response, and nutritional status—in decision-making processes in emergency abdominal surgery has been increasingly emphasized [12,13,14]. Albumin-based nutritional indices may be valuable in risk stratification because they reflect not only nutritional status but also inflammatory burden. Recent studies on emergency abdominal surgery have revealed that these indices can be helpful in selecting the surgical approach and predicting postoperative morbidity and mortality [12,13,14]. Current guidelines emphasize that the diagnosis and management of serious pathologies such as complicated diverticulitis should be based not only on anatomical staging but also on blood parameters and physiological findings [15,16].

This study aimed to evaluate the predictive role of albumin-based nutritional indices in determining the surgical strategy (primary anastomosis or Hartmann procedure) in the preoperative period in patients treated operatively for complicated left-sided colonic diverticulitis.

## 2. Materials and Methods

This retrospective study evaluated patients who were operatively treated for complicated left colonic diverticulitis at Başakşehir Çam and Sakura City Hospital between August 2021 and August 2024.

Inclusion criteria were as follows:

- Being over 18 years of age,
- Having complete access to data from hospital databases or clinical records,
- Having undergone operative treatment for Hinchey stage III or IV diverticulitis localized in the left-sided colon.

Exclusion criteria were as follows:

- Patients who have been treated operatively for reasons other than Hinchey stage III-IV (e.g., ileus, bleeding, fistula, unresponsiveness to conservative treatment),
- Surgical techniques other than resection and anastomosis (without diverting ileostomy) and the Hartmann procedure,
- Cases whose final pathology report is not compatible with diverticulitis.

**Figure 1:**
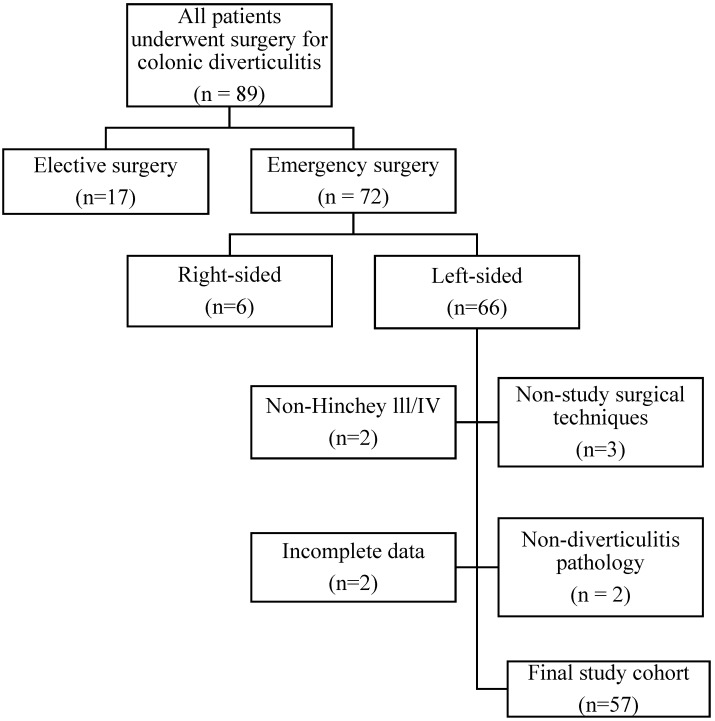
Flowchart of the patient inclusion and exclusion process for the study cohort.

Fifty-seven patients who matched the specified criteria were included in the study (Figure 1). Ethical approval for this study was obtained from the Başakşehir Çam and Sakura City Hospital Scientific Research Ethics Committee No. 2 (Approval Code: 2024-06, Approval Date: 6 September 2024). The study was conducted in accordance with the principles outlined in the Helsinki Declaration. Although the study had a retrospective design, informed consent was routinely obtained from patients, allowing their data to be used in scientific studies in accordance with institutional protocol.

Demographic characteristics, comorbidities, clinical data, Prognostic Nutrition Index (PNI), Hemoglobin–Albumin–Lymphocyte–Platelet (HALP) score, CRP–Albumin–Lymphocyte (CALLY) index and, Modified Glasgow Prognostic Score (mGPS) of the patients included in the study were evaluated. Additionally, intraoperative findings, details of surgical techniques, and postoperative complications were analyzed.

The patients were divided into two groups based on the surgical method—those who underwent the Hartmann procedure and those who underwent primary anastomosis (without diverting ileostomy)—and all analyses were performed comparatively between these two groups. Albumin-based nutritional indices were calculated as follows:

PNI = (Albumin [g/L]) + (5 × Lymphocyte count [109/L])

CALLY Index = (Lymphocyte count [109/L] × Albumin [g/L])/CRP [mg/L]

mGPS:

Score 2: CRP > 10 mg/L and Albumin < 35 g/L

Score 1: CRP > 10 mg/L and Albumin ≥ 35 g/L

Score 0: CRP ≤ 10 mg/L (regardless of albumin level)

HALP = (Hemoglobin [g/dL] × Albumin [g/L] × Lymphocyte count [109/L])/Platelet count [109/L]

Among comorbid conditions, the presence or absence of diabetes mellitus, pulmonary disease, cardiovascular disease, and renal disease was analyzed separately. Body mass index (BMI) was calculated by dividing body weight in kilograms by the square of height in meters (kg/m^2^). Hinchey staging was based on intraoperative findings; purulent peritonitis was classified as Hinchey stage III, and fecal peritonitis as Hinchey stage IV. Postoperative complications were graded according to the Clavien–Dindo classification (CDC) and categorized into two groups in the analyses: CDC stage ≥ 3 and <3.

### 2.1. Surgical Management

After admission to the emergency department, all patients stopped oral intake and started intravenous hydration to eliminate fluid volume deficiency. All patients were started on intravenous empirical antibiotic therapy effective against Gram-negative bacilli and anaerobic organisms. The clinical severity of the disease determined the choice of antibiotic agent.

The decision for operative management was made by the senior surgeon based on the patient’s clinical status, laboratory findings, and imaging results. The surgical method and technique were determined based on the experience of the surgical team and the patient’s current clinical condition. In patients who underwent primary resection and anastomosis, the anastomosis was performed in a double layer with a stapler or manually, depending on the surgeon’s preference.

During the postoperative follow-up, oral feeding was targeted for patients as early as possible. In case of clinical necessity, the empirical antibiotic treatment was revised. Patients were discharged after complete clinical relief.

### 2.2. Statistical Analysis

All statistical analyses were performed using the IBM SPSS Statistics for Windows, Version 25.0 (IBM Corp., Armonk, NY, USA) program. The conformity of continuous variables to normal distribution was evaluated with the Kolmogorov–Smirnov test. Mann–Whitney U test was applied for non-normally distributed data, and Student’s *t*-test was used for normally distributed data. Categorical variables were presented as numbers (n) and percentages (%), and continuous variables were presented as mean ± standard deviation in parametric data and as median (interquartile range, IQR) in nonparametric data. The Pearson chi-square (χ^2^) test was used to compare categorical variables.

Receiver Operating Characteristic (ROC) analysis was performed to determine the cut-off value for statistically significant metric variables. In addition, to determine independent predictors of the Hartmann procedure, the variables found to be significant were included in the multivariate logistic regression analysis with the forward stepwise method. The significance level was accepted as *p* < 0.05 in all statistical tests.

## 3. Result

A total of 57 patients were included in the study. The overall mean age of the patients was 63.1 ± 16.12 years, and 35 (61.4%) were male. When grouped according to surgical technique, 17 patients (29.8%) underwent primary anastomosis and 40 (70.2%) underwent Hartmann procedure. The mean age in the Hartmann procedure group was significantly higher than in the primary anastomosis group (65.93 ± 14.09 vs. 56.47 ± 18.98 years; *p* = 0.042). No significant differences were found between the groups in terms of gender, diabetes mellitus, pulmonary disease, heart disease, and renal disease (*p* > 0.05). No difference was found between the two groups in terms of BMI (*p* = 0.176). However, the systemic inflammatory response syndrome (SIRS) rate was significantly higher in the Hartmann procedure group (55% vs. 11.76%; *p* = 0.002).

When laboratory parameters were evaluated, CRP levels were significantly higher in the Hartmann group [153.50 mg/L (56.13–223.75) vs. 29.20 mg/L (17.75–147.10); *p* = 0.002], and albumin levels were significantly lower (33.68 ± 6.62 g/L vs. 41.24 ± 4.02 g/L; *p* < 0.001). Similarly, the lymphocyte count was significantly lower in the Hartmann group [1.12 (0.70–1.46) vs. 1.80 (0.79–2.49); *p* = 0.02]. There was no significant difference between the groups in terms of hemoglobin levels, platelet count, and operative time (*p* > 0.05). Although the Hinchey stage IV diverticulitis rate was higher in the Hartmann group, the difference was not statistically significant (72.5% vs. 52.94%; *p* = 0.152). Although the postoperative major complication rates (Clavien–Dindo stage ≥ 3) were higher in the Hartmann group, the difference did not reach the limit of statistical significance (27.5% vs. 11.76%; *p* = 0.195) (Table 1).

In the evaluation of preoperative albumin-based nutritional indices, which constitute the study’s primary focus, PNI, HALP score, and CALLY index were found to be statistically significantly lower in patients who underwent the Hartmann procedure. PNI (39.07 ± 8.19 vs. 49.86 ± 7.88; *p* < 0.001), HALP score [1594.44 (860.13–2399.7) vs. 3365.37 (1442.51–5781.17); *p* = 0.018], and CALLY index [0.21 (0.11–0.47) vs. 1.02 (0.44–4.24); *p* < 0.001] were significantly lower in the Hartmann group compared to the primary anastomosis group. In the mGPS evaluation, 57.5% of the patients in the Hartmann group had an mGPS score of 2, while this rate was only 5.88% in the primary anastomosis group (*p* < 0.001) (Table 2).

**Figure 2:**
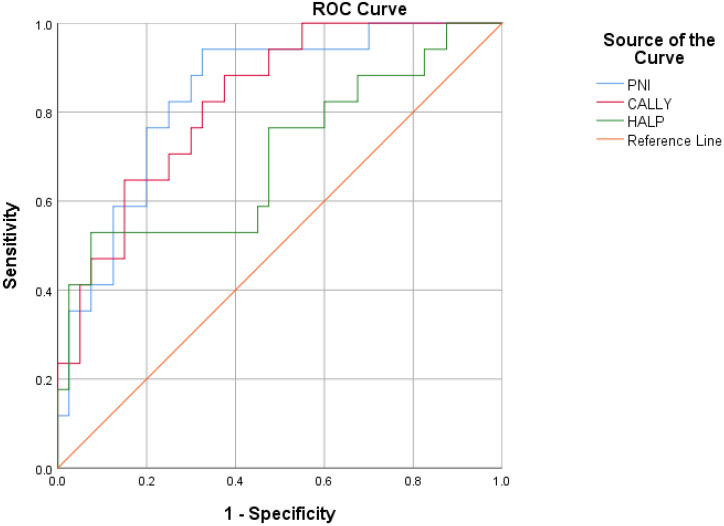
ROC Curves of PNI, CALLY, and HALP score.

According to the ROC analysis results, albumin, PNI, and CALLY index stood out as the parameters with the highest AUC values. The AUC for albumin level was 0.835 (*p* < 0.001), while with a cut-off value of 38.5 g/L, sensitivity was calculated to be 76.5% and specificity 80.0%. The AUC for PNI was 0.840 (*p* < 0.001), and 76.5% sensitivity and 77.5% specificity were obtained at a cut-off value of 43.22. The AUC for the CALLY index was 0.826 (*p* < 0.001), with a cut-off value of 0.45; sensitivity was 70.6% and specificity was 72.5%. In addition, CRP (AUC: 0.757; *p* = 0.002; cut-off value: 68.5 mg/L; sensitivity: 70.0%; specificity: 70.6%), lymphocyte count (AUC: 0.696; *p* = 0.02; cut-off: 1.31; sensitivity: 64.7%; specificity: 67.5%) and HALP score (AUC: 0.700; *p* = 0.018; cut-off: 1800.98; sensitivity: 58.8%; specificity: 55.0%) were also found to have significant predictive value (Figure 2, Table 3).

Finally, multivariate logistic regression analysis was performed using the forward stepwise method. In the third step of the analysis, albumin level < 38.5 g/L (OR: 16.53; 95% CI: 2.877–95.066; *p* = 0.002), SIRS detection (OR: 12.98; 95% CI: 1.582–111.111; *p* = 0.017), and CALLY index < 0.45 (OR: 6.40; 95% CI: 1.136–36.158; *p* = 0.035) were determined as independent predictors of the Hartmann procedure (Table 3).

## 4. Discussion

In this study, we aimed to evaluate the predictive role of albumin-based nutritional indices in the decision of surgical techniques in patients treated operatively for complicated left colonic diverticulitis. In the operative management of diverticular disease, surgical technique decisions are often shaped by the patient’s clinical condition and the surgeon’s experience. However, this decision-making process cannot always be based on objective criteria. Systemic inflammation and nutritional status are associated with surgical prognosis; therefore, we believe that albumin-based nutritional indices assessed preoperatively may guide surgical technique decisions. Although such indices alone are not sufficient to decide on surgical technique, they can help us make more accurate and individualized decisions in patient management. Albumin-based nutritional indices, such as the CALLY index, HALP score, PNI, and mGPS, have been evaluated in various subjects in the literature; however, their effects on survival in cancer patients have been examined more comprehensively [17,18,19,20]. Studies evaluating these biomarkers for diverticulitis are quite limited in the literature [21,22,23]. There are no studies in the literature evaluating the effect of surgical technique on the decision-making process, especially in patients treated operatively for complicated diverticulitis; in this respect, our study is the first original study in this field.

Our study identified albumin level < 38.5 g/L, SIRS detection, and CALLY index < 0.45 as independent risk factors for the Hartmann procedure. It is also reported in the literature that the Hartmann procedure is preferred more frequently in septic and malnutritional patients [24,25]. The higher rate of SIRS detection in the Hartmann procedure group is closely related to the reasons for choosing this surgical procedure. In our study, the Hartmann procedure was generally preferred in more septic, physiologically unstable, or high-risk patients. In this context, SIRS detection is not a predictor of the Hartmann procedure, but rather a reflection of the severity of the disease that leads to the choice of this technique. Therefore, a high inflammatory response should not be considered a direct predictor of the Hartmann procedure but rather a natural part of the patient profile for which this technique is preferred. Similarly, in the literature, it is emphasized that septic status or severe physiological deterioration are important factors limiting the decision for primary anastomosis [24,25]. For this reason, the Hartmann procedure stands out as a safe and life-saving surgical option in patients with systemic inflammation and hypoproteinemic course. For this reason, the Hartmann procedure stands out as a safe and life-saving surgical option in patients with systemic inflammation and hypoproteinemia. In our study, the cut-off value for albumin was determined to be 38.5 g/L; in the ROC analysis, this value had an AUC of 0.835 (*p* < 0.001), with 76.5% sensitivity and 80.0% specificity.

The CALLY index is a multiparameter score that evaluates inflammation, nutrition, and immune response in malignancies and different clinical conditions [13]. This index provides a holistic assessment of the patient’s overall physiological status by reflecting the inflammatory response through CRP level, immune capacity through lymphocyte count, and nutritional status through albumin level. Our study is the first in the literature to evaluate the CALLY index to predict surgical technique decisions in cases of complicated left-sided colonic diverticulitis. According to our findings, the Hartmann procedure preference was 6.4 times higher in patients with a CALLY index of less than 0.45. This index was generally used in previous studies to predict survival or postoperative complications in gastrointestinal system malignancies [17,26]. In a study by Angın et al. [13], the capacity of this score to predict bowel ischemia in abdominal wall hernias, a non-malignant condition, was evaluated, and a relationship was found between low CALLY scores and bowel ischemia. The CALLY index, which combines CRP, albumin, and lymphocyte levels into a single score, was found to be effective in predicting surgical technique decisions rather than complications, and this is a unique and clinically valuable contribution of our study to the literature. 

mGPS is calculated using albumin and CRP values [20]. These parameters are biomarkers routinely evaluated in the preoperative period, and it is extremely easy to calculate this score. In this respect, mGPS is an advantageous score in terms of integration into clinical practice. Although mGPS showed a statistically significant difference between the two groups in our study, it was not identified as an independent predictor for the Hartmann procedure in multivariate analysis. This suggests that mGPS may have limited predictive power in terms of surgical technique decisions in complicated diverticulitis. Park et al. [21] reported that mGPS had no predictive value for conservative treatment failure in patients with right colonic diverticulitis. Angın et al. [13] evaluated the role of mGPS in predicting bowel ischemia in strangulated abdominal wall hernias; however, no significant difference was found. These data reveal that mGPS does not fully reflect the severity of diverticulitis and ischemic diseases and may not be sufficient to guide surgical decision-making processes.

PNI is based on the combination of lymphocyte count and albumin level and provides information about the patient’s nutritional status and immunological response. PNI has been evaluated in various clinical scenarios in the literature, and studies related to malignancy are particularly prominent [19,20]. In our study, although there was a significant difference between the groups in terms of PNI value, it was not found to be an independent predictor of the Hartmann procedure in multivariate analysis. A study by Canlıkarakaya et al. [23] reported that PNI was not associated with complicated diverticulitis. In the literature, data on the PNI for predicting the severity of pancreatitis are inconsistent [27,28]. These results suggest that PNI may not be a determining factor in the decision-making process for surgical technique.

The HALP score is a composite biomarker that combines albumin, hemoglobin, lymphocyte, and platelet values. Although more complex to calculate compared to other parameters, a low HALP score is reported to be associated with increased operative treatment rate and mortality in patients with ileus [14]. However, in our study, the HALP score could not be shown to have a predictive role in the decision-making process of surgical technique. Benli et al. [12] found a significant association between low HALP scores and the development of complications and postoperative morbidity in patients with acute appendicitis. These different clinical results suggest that the HALP score cannot be generalized independently of etiology in inflammation-related surgical diseases and should be evaluated individually in each clinical situation.

On the other hand, the absence of a statistically significant difference in Hinchey stage IV rates between the groups in our study supports the basic message of our study. Current guidelines recommend that the surgical technique decision in patients with complicated diverticulitis should be based not only on the anatomical stage but also on the patient’s clinical condition and comorbidities [15,16]. We believe that the most appropriate approach is to base surgical technique decisions not only on anatomical staging, but also on a comprehensive assessment of the patient’s preoperative physiological status, comorbidities, blood parameters, and overall clinical picture. At this point, due to the retrospective nature of our study, it is not possible to individually determine whether the chosen surgical technique is the most appropriate decision for each patient. However, in all cases, the surgical technique decision process was made by evaluating the patient’s condition holistically. During this process, treatment algorithms were individualized on a patient-by-patient basis by the surgical team. In addition, only patients with complicated diverticulitis (Hinchy stage III and IV) were included in our study, and current guidelines recommend that the surgical technique decision in this patient group should be made by the patient-based surgical team [15]. Therefore, our study reflects the decision-making processes applied in real-life practice and the individualized approach based on the surgeon’s clinical assessment, while also revealing the potential guiding role of objective parameters used in the preoperative period in this decision-making process.

Our study has some limitations. First, due to the retrospective design of the study, the possibility of bias in sample selection cannot be eliminated. In addition, the fact that it was conducted in a single center with a limited number of patients limits the generalizability of the results obtained. However, surgeon-dependent subjective decisions regarding surgical techniques may introduce variability, preventing full standardization of some variables. Additionally, the study did not evaluate ostomy reversal rates and long-term clinical outcomes. Consideration of these parameters in multicenter prospective studies will contribute to more precise positioning of the CALLY index in surgical decision-making processes.

## 5. Conclusions

Our study is the first to evaluate albumin-based nutritional indices in predicting surgical technique selection in patients treated operatively for complicated left-sided colonic diverticulitis. Our findings revealed that SIRS detection and a low CALLY index were independent predictors for the Hartmann procedure. The CALLY index provides a holistic assessment of the patient’s overall physiological status by reflecting the inflammatory response, immune capacity, and nutritional status. The CALLY index can assist surgeons in making more objective and personalized decisions, and its integration into clinical practice has the potential to improve patient outcomes. However, multicenter and prospective studies are needed to evaluate the generalizability of the findings and their applicability in routine clinical practice.

Take-Home Messages:

- A CALLY index < 0.45 was found to be an independent predictor of the Hartmann procedure in patients treated operatively for complicated left colonic diverticulitis.
- The CALLY index is a practical index that combines CRP, albumin, and lymphocyte levels to comprehensively reflect the inflammatory response, immune status, and nutritional status.
- Integration of this index into clinical decision processes may assist surgeons in more objective and personalized surgical technique decisions.
- In addition, the study found that the Hartmann procedure was more frequently chosen in patients with albumin levels < 38.5 g/L and detected SIRS.
- The findings indicate that not only anatomical staging but also the patient’s physiological status and laboratory parameters should be taken into account when deciding on the surgical technique.

## Figures and Tables

**Table 1:** Comparison of clinical, laboratory, and preoperative albumin-based nutritional indices between primary anastomosis and Hartmann procedure groups.

	Primary Anastomosis (n = 17)	Hartmann’s Procedure (n = 40)	*p* Value
Male sex ^β^	10 (58.52)	25 (62.5)	0.794
Age ^α^ (years)	56.47 ± 18.98	65.93 ± 14.09	0.042
BMI ^Ω^ (kg/m^2^)	26.44 ± 3.97	28.17 ± 4.51	0.176
Diabetes mellitus ^β^ (Yes)	1 (5.88)	6 (15)	0.337
Pulmonary disease ^β^ (Yes)	1 (5.88)	11 (27.5)	0.067
Heart disease ^β^ (Yes)	3 (17.64)	16 (40)	0.101
Renal disease ^β^ (Yes)	3 (17.64)	3 (7.5)	0.253
SIRS ^β^ (Yes)	2 (11.76)	22 (55)	0.002
Hemoglobin ^α^ (g/dL)	12.89 ± 2.04	12.63 ± 2.39	0.692
Platelet ^Ω^ (10^9^/L)	300 (251.5–325.5)	265 (227–345)	0.595
Lymphocyte ^Ω^ (10^9^/L)	1.80 (0.79–2.49)	1.12 (0.70–1.46)	0.02
CRP ^Ω^ (mg/L)	29.20 (17.75–147.10)	153.50 (56.13–223.75)	0.002
PNI ^α^	49.86 ± 7.88	39.07 ± 8.19	<0.001
HALP ^Ω^	3365.37 (1442.51–5781.17)	1594.44 (860.13–2399.7)	0.018
CALLY ^Ω^	1.02 (0.44–4.24)	0.21 (0.11–0.47)	<0.001
mGPS ^β^ (Score 2)	1 (5.88)	23 (57.5)	<0.001
Albumin ^α^ (g/L)	41.24 ± 4.02	33.68 ± 6.62	<0.001
Hinchey ^β^ (Stage IV)	9 (52.94)	29 (72.5)	0.152
Operative time ^α^ (min)	150.59 ± 51.35	156.05 ± 58.06	0.738
CDC ^β^ ≥ 3	2 (11.76)	11 (27.5)	0.195

^β^: numbers (%), ^α^: mean ± standard deviation, ^Ω^: median (IQR), BMI: body mass index, SIRS: systemic inflammatory response syndrome, CRP: C reactive protein, PNI: Prognostic Nutritional Index, HALP: Hemoglobin–Albumin–Lymphocyte–Platelet Score, CALLY: CRP–Albumin–Lymphocyte Index, mGPS: Modified Glasgow Prognostic Score, CDC: Clavien–Dindo classification.

**Table 2:** ROC analysis for metric variables with statistically significant differences.

	AUC (95% CI)	*p* Value	Cut-Off Value	Sensitivity (%)	Specificity (%)
Age (years)	0.646 (0.471–0.820)	0.084	63.5	55.0	58.8
CRP (mg/L)	0.757 (0.606–0.907)	0.002	68.5	70.0	70.6
Albumin (g/L)	0.835 (0.730–0.939)	<0.001	38.5	76.5	80.0
Lymphocyte (10^9^/L)	0.696 (0.523–0.869)	0.02	1.31	64.7	67.5
PNI	0.840 (0.732–0.948)	<0.001	43.22	76.5	77.5
HALP	0.700 (0.538–0.862)	0.018	1800.98	58.8	55.0
CALLY	0.826 (0.718–0.935)	<0.001	0.45	70.6	72.5

AUC: area under curve, CRP: C reactive protein, PNI: Prognostic Nutritional Index, HALP: Hemoglobin–Albumin–Lymphocyte–Platelet Score, CALLY: CRP–Albumin–Lymphocyte Index.

**Table 3:** Multivariate logistic regression analysis for independent predictors of Hartmann procedure.

	OR	95% CI	*p* Value
Albumin (<38.5 g/L)	16.53	2.877–95.066	0.002
SIRS (Yes)	12.98	1.582–111.111	0.017
CALLY (<0.45)	6.40	1.136–36.158	0.035

## Data Availability

The original contributions presented in the study are included in the article, further inquiries can be directed to the corresponding author.
